# A NIM PET/CT phantom for evaluating the PET image quality of micro-lesions and the performance parameters of CT

**DOI:** 10.1186/s12880-021-00683-4

**Published:** 2021-11-08

**Authors:** Shujie Lu, Peng Zhang, Chengwei Li, Jie Sun, Wenli Liu, Pu Zhang

**Affiliations:** Center for Medical Metrology, National Institute of Metrology, Beijing, China

**Keywords:** PET/CT phantom, CT performance parameters, Image quality, Micro-lesions

## Abstract

**Background:**

The commonly used NEMA IEC Body phantom has a number of defects, hindering its application for detecting micro-lesions and measuring the performance parameters of computed tomography (CT). This study aimed to propose a PET/CT phantom designed by National Institute of Metrology (NIM), China, which is capable of simultaneously testing the performance of PET and CT systems, and to evaluate the quality of imaging.

**Methods:**

The phantom developed in the present study, the NIM PET/CT phantom, is composed of a PET imaging module and a CT imaging module, and these modules are connected together through bolts, which can simultaneously measure the imaging performance of PET and CT systems. Hot spheres were filled with 4:1 sphere-to-background activity concentration using ^18^F-fluorodeoxyglucose (^18^F-FDG), and cold spheres were filled with non-radioactive water. We compared the results of imaging obtained from the NIM PET/CT phantom and the NEMA IEC Body phantom to assess their diagnostic efficacy. In order to evaluate the generalization ability of the NIM PET/CT phantom, three different PET/CT systems were used to scan on the same scanning protocol. To evaluate the effects of image reconstruction algorithms on image quality assessment, ordered subset expectation maximization (OSEM), OSEM-point-spread function (PSF), OSEM-TOF, and OSEM-PSF-TOF algorithms were employed.

**Results:**

The imaging quality of the NIM PET/CT phantom and the NEMA IEC Body phantom was relatively consistent. The NIM PET/CT phantom could detect 7 mm spheres without influencing the imaging quality. It was found that PSF reconstruction exhibited to reduce the speed of convergence, the contrast and background variability of spheres (13–28 mm) were significantly improved after two iterations. In addition to improve the image contrast and background variability, TOF could markedly improve the overall image quality and instrument detection limit. TOF-PSF could noticeably reduce noise level, enhance imaging details, and improve quality of imaging.

**Conclusions:**

The results showed that in comparison with the NEMA IEC Body phantom, the NIM PET/CT phantom outperformed in evaluating the PET image quality of micro-lesions and the performance parameters of CT.

## Background

Positron emission tomography (PET) has proven invaluable in the diagnosis, staging, and treatment response evaluation of a broad range of tumors [1]^.^ The technologies adopted and refined in PET systems mainly address basic imaging parameters, such as resolution, sensitivity, and aperture, influencing the overall quality of PET images [2–4]. X-ray computed tomography (CT) is based on differential absorption of X-ray by different tissues to enable distinction between different anatomical structures, and the CT uses sophisticated mathematical techniques to construct a two-dimensional (2D) image [1]. With the development of medical imaging techniques, medical image fusion emerged as the process of coalescing multiple images from multiple imaging modalities to obtain a fused image with a large amount of information for increasing the clinical applicability of medical images [2]. In initial staging, PET/CT exhibited a higher sensitivity in detecting distant metastases compared to conventional imaging, leading to disease upstaging and the consequent switch from a local approach to a systemic chemotherapy [5].

In medical imaging, physical phantoms refer to real objects designed to simulate the human body for specific clinical conditions. Physical phantoms are used to calibrate imaging systems, evaluate their performance, and ensure the correct operation of imaging systems before scanning human subjects. They also constitute an inexpensive way of testing new imaging applications and serve as a well-defined reference for quantitative measurements. Because of the differences in contrast from one region to another, images typically show a clear delineation of internal structure (anatomy), morphology, and physiological functions [6–8]. In 1994, the National Electrical Manufacturers Association (NEMA) published the NEMA NU2-1994 standard for performance assessment of PET. Thereafter, the NEMA NU2 standard and the IEC61675-1 standard issued by the International Electrotechnical Commission (IEC) have been repeatedly revised. According to the NEMA NU 2–2012 standard, image quality parameters of PET scanners could be obtained by measuring a specific IEC-61675–1 emission phantom. This image quality phantom mimics the shape of an upper human body and is built of acrylic glass. The PET component of the PET/CT system can be evaluated using the method described in the NEMA standard, and the CT component is mainly associated with the low-contrast resolution [8, 9].

Although PET-NEMA/IEC body phantom is widely used, it still has a number of shortcomings. Firstly, the minimum inner diameter of spheres used to measure image quality parameters in phantom is 10 mm, thus, the phantom cannot meet the requirements of detecting micro lesions [10]. Secondly, the mentioned phantom can only be applied to PET measurement. The third limitation is the requirement of specific temperature and humidity conditions for storage, to ensure the standardized use of phantom for repeated measurements and in multicenter trials. Hence, the present study aimed to propose a PET/CT phantom designed by National Institute of Metrology(NIM), China, which is capable of simultaneously testing the performance of PET and CT systems, and to evaluate quality of imaging.

## Methods

### The NEMA IEC body phantom

The NEMA IEC Body phantom [8] is an anthropomorphic phantom recommended by both NEMA and IEC, and it is extensively utilized in PET imaging. This phantom mimics the shape of an upper human body and is built of acrylic glass material. It comprises 6 hollow glass spheres (inner diameters of 37, 28, 22, 17, 13, and 10 mm), which can be inserted into the large phantom compartment. Additionally, a cylindrical insert containing styrofoam with an average density of 0.3 ± 0.1 g/ml, and is positioned in the center of the phantom [8, 11, 12].

**Fig. 1:**
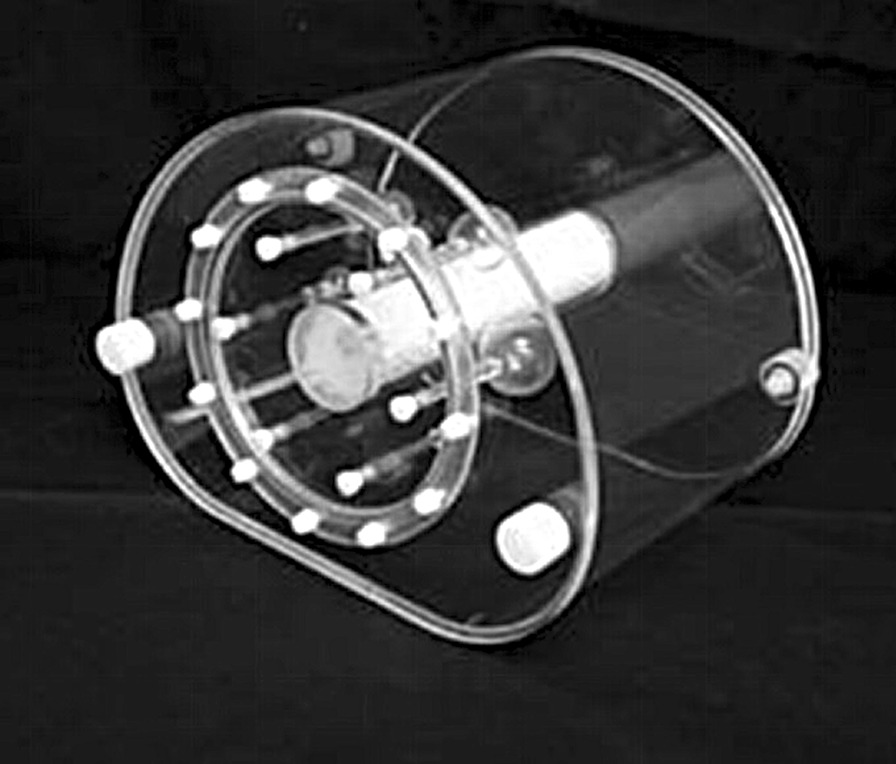
The NEMA IEC Body phantom

The NEMA IEC Body phantom simulates hot and cold lesions and reflects the clinical image quality of a PET/CT system. It two largest spheres (diameters of 37 and 28 mm) as the cold lesions and the other spheres (diameters of 22, 17, 13, and 10 mm) as the hot lesions. Besides, a cylindrical lung insert, filled partly with a low atomic number material with an average density of 0.30 ± 0.10 g/cc was used (Fig. 1).

### The NIM PET/CT phantom

**Fig. 2:**
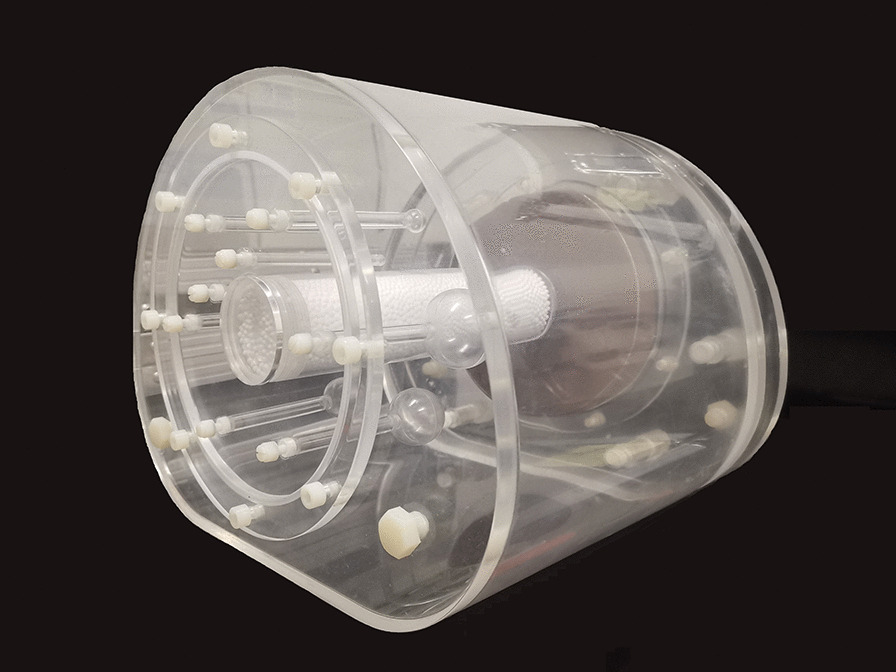
The NIM PET/CT phantom

The phantom developed in the present study is composed of a PET imaging module and a CT imaging module, and these modules are connected together through bolts. This phantom can simultaneously assess the imaging performance of PET and CT systems (Fig. 2).

**Fig. 3:**
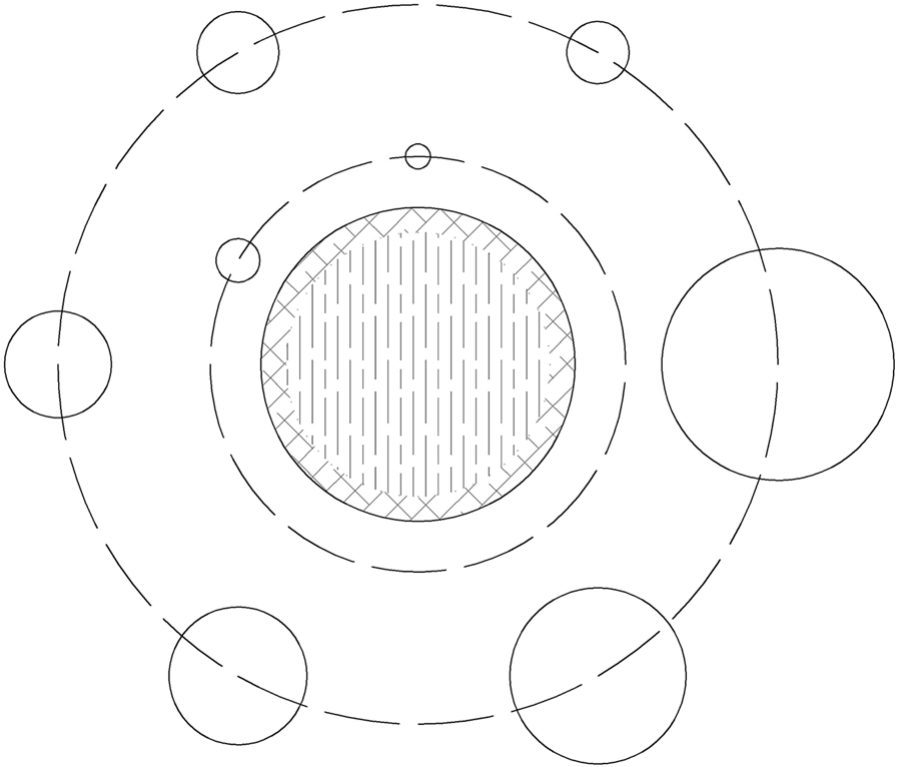
The distribution of spheres in the NIM PET/CT phantom

The structure of a PET imaging module in the NIM PET/CT phantom was found similar to that of the NEMA IEC Body phantom, in which filling was performed by ^18^F-fluorodeoxyglucose (F-FDG) solution. ^18^F-FDG, realizing the evaluation of glucose metabolism, is the most commonly used tracer in oncology because of the practical half-life of ^18^F (110 min) compared with other short-lived positron emitters [13]. In addition to the 6 hollow spheres specified in the NEMA NU2 standard, 2 hollow spheres with diameters of 4 and 7 mm were added. Among them, spheres with diameters of 28 and 37 mm were filled with purified water for cold lesions, and the remaining were filled with ^18^F-FDG solution for hot lesions. The distribution of spheres in the proposed phantom is shown in Fig. 3.

The CT imaging module was developed with a diameter of 150 mm and a thickness of 20 mm. The background was filled with purified water, and was made of a non-metallic CT-free artifact material, which was equivalent to water. Besides, three low-contrast CT inserts with the same diameter were inserted, with a triangle distribution, whose low-contrast resolution was 0.5%, 1.0% and 1.5%, respectively.

### Image quality control

#### Region of interest (ROI)

ROIs were placed on PET and CT images. ROIs of PET images included cold spherical ROIs, hot spherical ROIs, spherical ROIs, and lung insert ROI; ROIs of CT images involved internal ROIs and background ROIs.

Twelve ROIs (37 mm) are drawn on the background region. Background ROIs for spheres with diameters of 10, 13, 17, 22, and 28 mm are drawn concentric to the ROIs (37 mm) as indicated in the top background ROI.

**Fig. 4:**
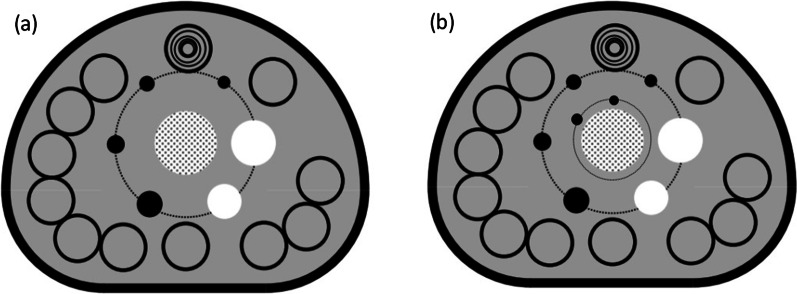
The layout of background ROIs for PET image quality analysis in (**a**) NEMA IEC Body phantom and (**b**) NIM PET/CT phantom

**Fig. 5:**
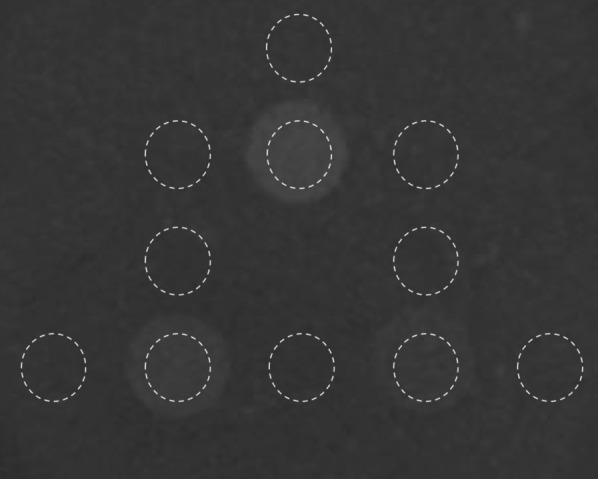
The layout of background ROIs for CT image quality analysis

All spherical ROIs were drawn on slices centered on the spheres and background ROIs with the same size and concentric distribution was on the background at the same level. Besides, ROIs were drawn on slices as close as possible to ± 1 and ± 2 cm on each side of the central slice. A total of 60 background ROIs, including 12 ROIs on each of five slices, were accordingly drawn (Fig. 4). Lung ROIs were drawn in form of a circle with a diameter of 30 ± 2 mm on the center of the simulated lung tube. ROIs of CT images included internal ROIs of each insert and the same background ROIs (n = 3) around each insert (Fig. 5).

#### PET image analysis

The contrast recovery coefficient (CRC) is the percentage of measured net concentration normalized by the measured background concentration to true net concentration normalized by true background concentration. CRC provides information of how accurately the system reproduces the true activity concentration in a specific volume [14]. CRC for hot sphere *j* (*Q*_*H,j*_) was defined as follows [8]:

1 $$Q_{H,j} = \frac{{{{C_{H,j} } \mathord{\left/ {\vphantom {{C_{H,j} } {C_{B,j} }}} \right. \kern-\nulldelimiterspace} {C_{B,j} }} - 1}}{R - 1} \times 100{\text{\% }}$$

where ${C}_{H,j}$ is the average counts in the *j*th hot sphere ROI, ${C}_{B,j}$ is the average counts in the background ROI, $R$ is the true sphere-to-background activity concentration ratio, and subscript *j* is the number of hot spheres.

CRC for cold sphere *j* (*Q*_*C,j*_) was defined as follows [8]:

2 $$Q_{C,j} = \left( {1 - \frac{{C_{C,j} }}{{C_{B,j} }}} \right) \times 100\%$$

where ${C}_{C,j}$ is the average counts in the *j*th cold sphere ROI*.*

#### Percentage of background variability

The percentage of background variability (*N*_*j*_) for sphere *j* was calculated as follows [8]:

3 $$N_{j} = \left( {\frac{{SD_{B,j} }}{{C_{B,j} }}} \right) \times 100\%$$

where ${SD}_{B,j}$ is standard deviation (SD) of the activity concentration in the background ROI*.*

#### Measurement of the residual error using CT-based attenuation and scatter-corrected PET images

To measure the residual error using CT-based attenuation and scatter-corrected PET images, the relative error (ΔC_lung,i_) was calculated for each slice (i) by calculating the ratio of the average counts in the lung insert ROI to the average counts in the background ROIs. Percentage of misplaced counts in the lung insert (∆Clung), following the NEMA NU 2-2012 guidelines, was defined by Eq. (4) [8]:

4 $$\Delta C_{lung,i} = \frac{{C_{lung,i} }}{{C_{B,37mm} }} \times 100\%$$

where ${C}_{lung,i}$ is the average of the lung insert ROIs in slice *i*, and *C*_B, 37 mm_ is the average of the 60 background ROIs.

#### CT images with low-contrast resolution

CT images with a low-contrast resolution were presented by calculating differences in CT values, in which differences with 15, 10 and 5 HU between CT values indicated that1.5%, 1.0% and 0.5% of CT images had a low-contrast resolution, respectively.

The quantitative method of calculating contrast to noise ratio (CNR) took all the influence factors such as background material, targets and noise into consideration when used to evaluate low-contrast resolution. CNR for low-contrast resolution evaluation was calculated as follows [15]:

5 $$CNR \, = \, \left( {CT_{I} - \, CT_{B} } \right) \, / \, SD_{B}$$

where CT_B_ and CT_I_ are the mean CT values in background ROI and insert ROI respectively, and SD_B_ is the standard deviation in ROI_B_.

### Data acquisition

Hot spheres were filled with 4:1 sphere-to-background activity concentration using ^18^F-FDG, and cold spheres were filled with non-radioactive water for cold lesions. Place the phantom on the patient table after effective assembly, and scanning was started immediately after positioning. Whole-body spiral CT scan was performed with a matrix of 512 × 512, pixel size of 2.0 × 2.0 mm, a slice thickness of 2.00 mm, and a tube voltage of 120.00 kV. Besides, PET acquisition time was about 8 min (Table 1).

**Table 1:** Comparing the measurement parameters of the NIM PET/CT phantom and NEMA IEC Body phantom

	NIM PET/CT phantom	NEMA IEC body phantom
*C*_*B*_ (kBq/mL)	6.6	6.6
*C*_*H*_ (kBq/mL)	26.4	26.4
A (kBq)	62,900	64,750
T (min)	8	8

*C*_*H*_, the mean activity concentration in the hot spheres; *C*_*B*_, the mean activity concentration in the background; A, the total activity; T, PET imaging time

### Image reconstruction

Generally, the performance parameters of PET and the visibility of lesions in phantoms are strongly dependent on parameters of the reconstruction algorithms. In PET, positron range and inter-crystal scattering can affect measurement resolution, and each can be modeled within system matrix. However, the calculation of such factors within the system matrix impacts the speed of computation and the convergence of the algorithm. During investigation of novel scenarios in PET, the accuracy of the system matrix can be chosen to reflect the application of the system and the speed required during image reconstruction [11, 12]. All PET images were reconstructed with the matrix size of 256 × 256 and the voxel size of 2.44 × 3.66 × 2.44 mm^3^. The PET data were reconstructed using the ordered subset expectation maximization (OSEM) algorithm, and the number of iterations was 2 [16, 17]. The main motivation for time-of-flight (TOF)-PET has always been the potential image quality improvement or reduction in image acquisition time of TOF-PET. TOF-PET data can be easily compared with PET data for the same study as the TOF data can be ignored during reconstruction [18–20]. The point-spread function (PSF) reconstruction produces images with the improved isotropic spatial resolution, the reduced ratio of spill-in/spill-out, and the increased activity concentration in micro lesions that can be more easily detected and characterized [11, 18, 21]. In order to evaluate the effects of TOF-PET on image quality assessment, OSEM, OSEM-PSF, OSEM-TOF, and OSEM-PSF-TOF algorithms were employed [22]. The PET reconstruction conditions are showed in Table 2.

**Table 2:** PET reconstruction conditions

Phantom	Algorithm	The number of iterations	Matrix	Pixel size (mm)	Thickness (mm)
NIM PET/CT phantom	OSEM	2	256 × 256	2.44 × 3.66	2.44
	OSEM-PSF				
	OSEM-TOF				
	OSEM-PSF-TOF				
NEMA IEC body phantom	OSEM-PSF-TOF	2	256 × 256	2.44 × 3.66	2.44

## Results

### PET-dependent parameters for image quality assessment

Image quality assessment was undertaken for both cold and hot lesions with different sizes to provide an indicator for the detection of lesions. A number of factors, such as emission scan duration, ^18^F-FDG activity concentration, target-to-background activity concentration ratio, and body mass index of the scanned object can affect CRC and background variability, identified as PET image quality descriptors. The attenuation correction strategies can be divided into (a) methods based on image segmentation, (b) machine learning methods, and (c) data-driven approaches, utilizing PET data alone or in synergy with existing CT data. The lower the residual error is, the higher accuracy of attenuation correction of the PET/CT system is.

**Fig. 6:**
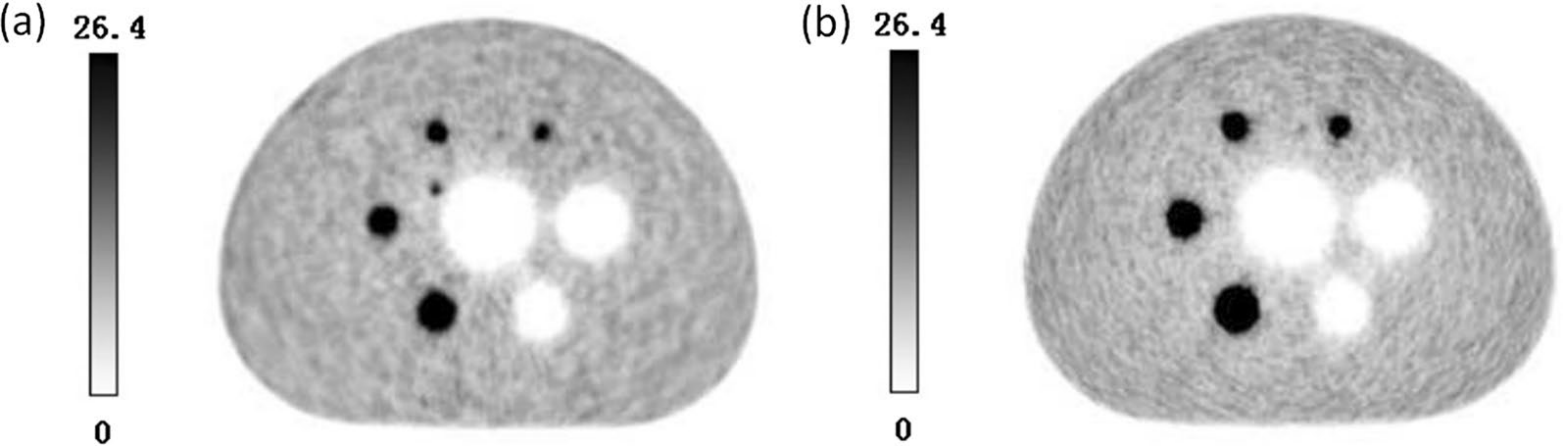
PET images of (**a**) NIM PET/CT phantom and (**b**) NEMA IEC Body phantom

The NIM PET/CT phantom and NEMA IEC Body phantom were used to assess the quality of PET images with the same PET/CT system and the same scanning protocol. Figure 6 shows a similar quality of PET images by both NIM PET/CT phantom and NEMA IEC Body phantom, indicating that the measurement results of the two phantoms would be comparable to some extent. Besides, the NIM PET/CT phantom was designed with 2 smaller spheres (4 and 7 mm), and a 7 mm sphere could be observed in the 10 o'clock direction of the phantom image, which could evaluate the imaging quality of PET/CT in micro lesions. As presented in Table 3, the NIM PET/CT phantom could measure the quality of PET images and accurately estimate the residual error using the CT-based attenuation and scatter-corrected PET images according to the current standards, and the added spheres slightly influenced the contrast(maximum deviation is 2.68%), background variability(maximum deviation is 0.32%), and residual error(deviation is 0.12%).

**Table 3:** Comparison of PET-dependent parameters between NIM PET/CT phantom and NEMA IEC Body phantom

	Sphere size (mm)	Contrast (%)	Background variability (%)	Residual error (%)
NIM PET/CT phantom	37	82.11	2.05	**8.98**
	28	75.81	2.67	
	22	73.11	3.27	
	17	71.43	3.86	
	13	61**.09**	**4.64**	
	10	45.08	5.46	
	7	20.52	6.45	
	4	–	–	
NEMA IEC Body phantom	37	82.21	1.99	**9.10**
	28	74.84	2.53	
	22	72.38	3.04	
	17	69.01	3.59	
	13	**58.41**	**4.32**	
	10	42.52	5.33	

Since the device could not detect 4 mm spheres, no measurement data of those spheres in the measurement module could be achieved. The maximum deviations are highlighted in bold

**Fig. 7:**
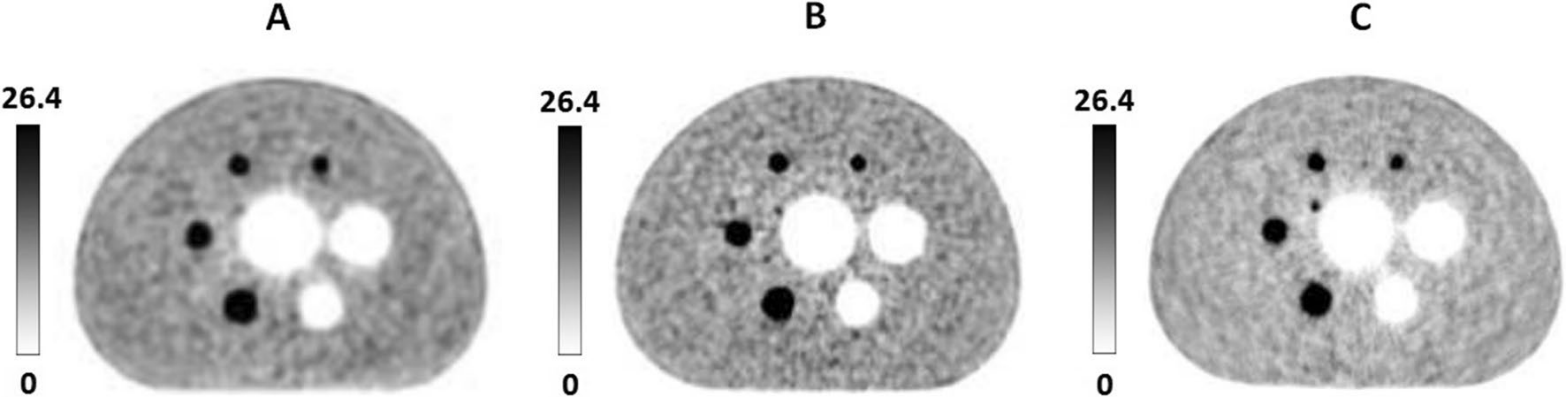
PET reconstruction images of the NIM PET/CT phantom on different systems

**Fig. 8:**
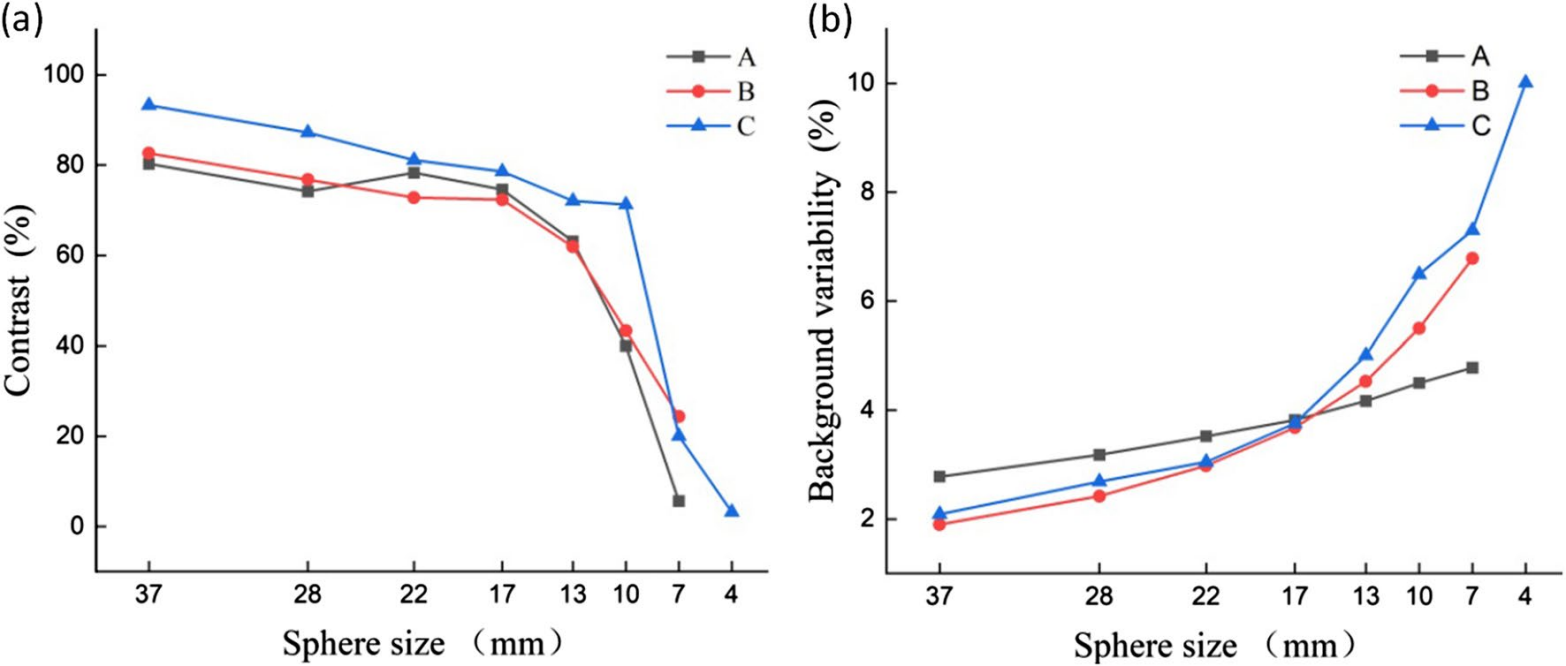
Comparing image quality among “A”, “B”, and “C” systems for (**a**) contrast and (**b**) background variability

In order to evaluate the generalization ability of the NIM PET/CT phantom, three different PET/CT systems were used to scan on the same scanning protocol. The results (Fig. 7) showed that these systems could detect a 7 mm sphere. The image contrast of “A” and “B” systems were similar in spheres larger than 10 mm, and the maximum deviation (5.48%) occurred in the 22 mm sphere. For 37 mm and 28 mm spheres, the “B” system presents better image quality than system “A”, while for (22–13) mm spheres that is opposite. However, this trend changed again when the spheres were directly less than 10 mm. The image quality of “C” system was higher than that of “A” and “B” systems, in which “C” system could further detect the 4 mm sphere, and the residual error was significantly lower than that of “A” and “B” systems (as illustrated in Table 4 and Fig. 8). It was found that the NIM PET/CT phantom could measure the image quality of micro-lesions in PET/CT, thereby significantly increasing the ability to evaluate different imaging systems to detect micro-lesions.

**Table 4:** Image quality data of the NIM PET/CT phantom on three PET/CT systems

PET/CT system	Sphere size (mm)	Contrast (%)	Background variability (%)	Residual error (%)
A	37	80.27	2.78	7.46
	28	74.14	3.18	
	22	78.28	3.52	
	17	74.59	3.82	
	13	63.12	4.17	
	10	39.95	4.5	
	7	5.61	4.78	
	4	–	–	
B	37	82.61	1.9	8.45
	28	76.75	2.42	
	22	72.8	2.98	
	17	72.3	3.68	
	13	61.9	4.53	
	10	43.26	5.5	
	7	24.38	6.78	
	4	–	–	
C	37	93.26	2.09	3.24
	28	87.21	2.69	
	22	81.12	3.05	
	17	78.54	3.76	
	13	72.08	5.01	
	10	71.27	6.49	
	7	20.06	7.3	
	4	3.22	10.01	

Neither A nor B system could detect 4 mm spheres, thus, data of 4 mm spheres could not be measured

### CT-dependent parameters for image quality assessment

**Fig. 9:**
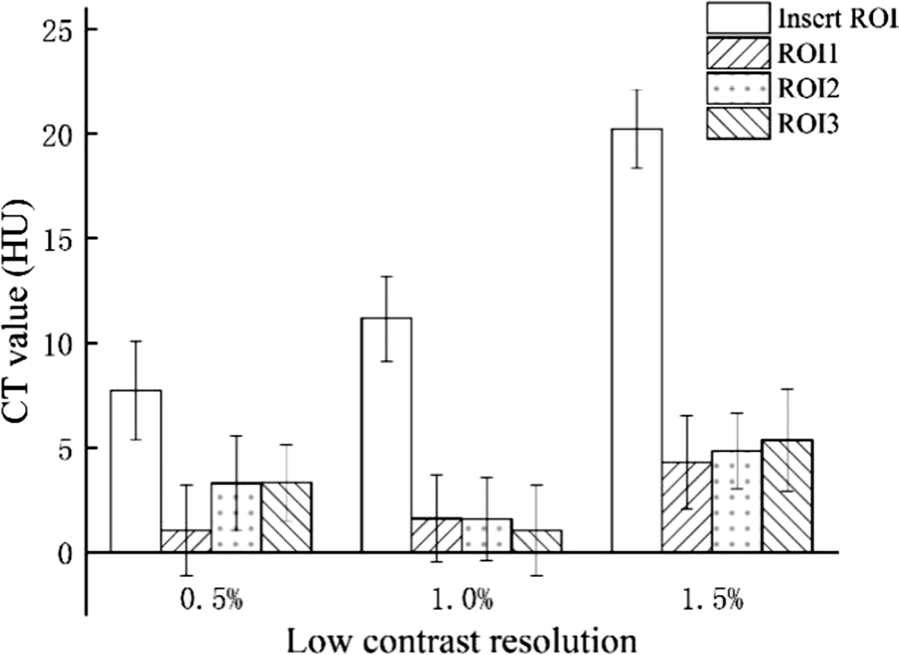
CT value versus low-contrast resolution

The low-contrast module of the NIM PET/CT phantom contained 3 inserts with the low-contrast resolution of 1.5%, 1.0% and 0.5%, respectively, which could meet the measurement requirements of different PET/CT systems. ROIs with an equal size were drawn inside and around each insert (Fig. 5), CT values within each ROI were measured, and then, differences in CT values, SD, and CNR were calculated (Table 5, Fig. 9). The results were in a good agreement with the truth values. The CT values and the SD values in the background ROIs around the same insert were similar, thus the background uniformity was satisfactory.

**Table 5:** Low-contrast resolution results of the CT scan

Low-contrast resolution	ROIs	Insert	Background #1	Background #2	Background #3
0.5%	CT value (HU)	7.73	1.04	3.30	3.32
	SD (HU)	2.37	2.18	2.28	1.83
	Mean of ***Δ***CT (HU)	**5.18**			
	CNR	2.47			
1.0%	CT value (HU)	11.16	1.61	1.57	1.04
	SD (HU)	2.03	2.08	1.98	2.18
	Mean of ***Δ***CT (HU)	**9.75**			
	CNR	4.69			
1.5%	CT value (HU)	20.23	4.29	4.85	5.36
	SD (HU)	1.89	2.24	1.80	2.44
	Mean of ***Δ***CT (HU)	**15.40**			
	CNR	7.25			

***Δ***CT = CT_Insert_ − CT_Background-i_ i = 1, 2, and 3. Numbers in bold are the low-contrast measurement results

**Fig. 10:**
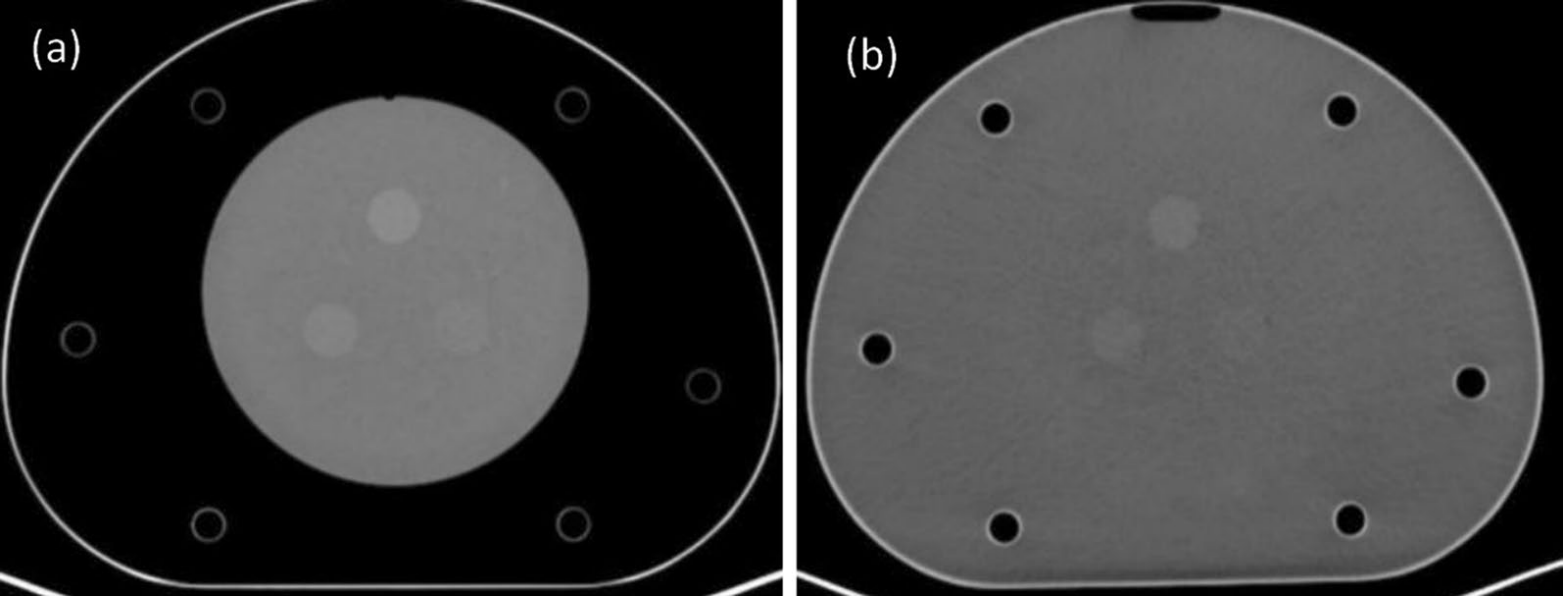
The verification of water-equivalent characteristic in the background of low-contrast CT module for injection of purified water. (**a**) Before, (**b**) after

**Fig. 11:**
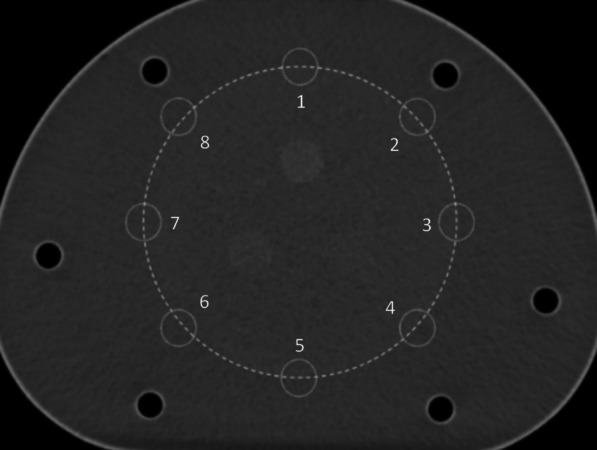
Noise measurement at the boundary between purified water and background. The circles 1–8 represent ROIs 1–8

Moreover, due to the good water-equivalent characteristic of the background materials, the background is almost integrated with purified water for injection (Fig. 10). In order to quantify the water-equivalent characteristic of the background, 8 ROIs with an equal size were selected to verify the imaging characteristic at the boundary between purified water and the background (Fig. 11), and the SD of CT value for each ROI was calculated (Table 6). According to the measurement results, the SD was relatively small (maximum SD is 3.65 HU as the bold number showed), indicating that the CT value smoothly varied at the boundary and degree of water-equivalence in the phantom was satisfactory.

**Table 6:** Results of noise measurement at the boundary between purified water and background

ROIs	ROI-1	ROI-2	ROI-3	ROI-4	ROI-5	ROI-6	ROI-7	ROI-8
SD (HU)	3.31	3.36	3.57	**3.65**	3.26	3.43	3.29	3.32

Number in bold is the maximum SD

**Fig. 12:**
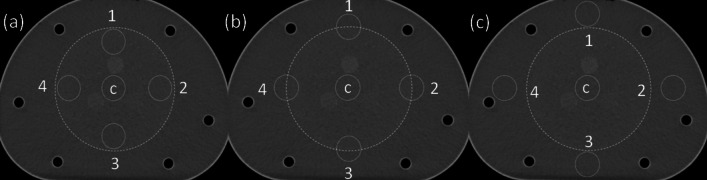
CT value acquisition for image uniformity for (**a**) background of CT module, (**b**) boundary between background and purified water, and (**c**) pure water area. The circles 1–4 represent ROIs 1–4, and the circle “c” represents “ROI-c”

To further understand the performance of an integrated CT system, CT values were respectively measured in the background and the boundary between the background and purified water (Fig. 12), and the CT values in the main ROI and in the four ROIs were recorded (Table 7). The CT values at the boundary were found similar and relatively stable, and the uniformity of CT under background, boundary and purified water mode were 2.07, 1.7, and 1.85, respectively.

**Table 7:** CT image uniformity

Location	Background		Boundary		Purified water	
ROIs	CT value (HU)	***Δ***CT value (HU)	CT value (HU)	***Δ***CT value (HU)	CT value (HU)	***Δ***CT value (HU)
ROI-c	4.51	0	4.51	0	4.51	0
ROI-1	6.1	1.59	6.21	**1.7**	3.74	0.77
ROI-2	5.15	0.64	5.77	1.26	2.88	1.63
ROI-3	6.46	1.95	4.88	0.37	3.2	1.31
ROI-4	6.58	**2.07**	5.78	1.27	2.66	**1.85**
Image uniformity	**2.07**		**1.7**		**1.85**	

***Δ***CT = CT_ROI-i_ − CT_ROI-c_ i = 1, 2, 3. Numbers in bold represent the CT image uniformity measurement results

### Comparison of image reconstruction algorithms

**Fig. 13:**
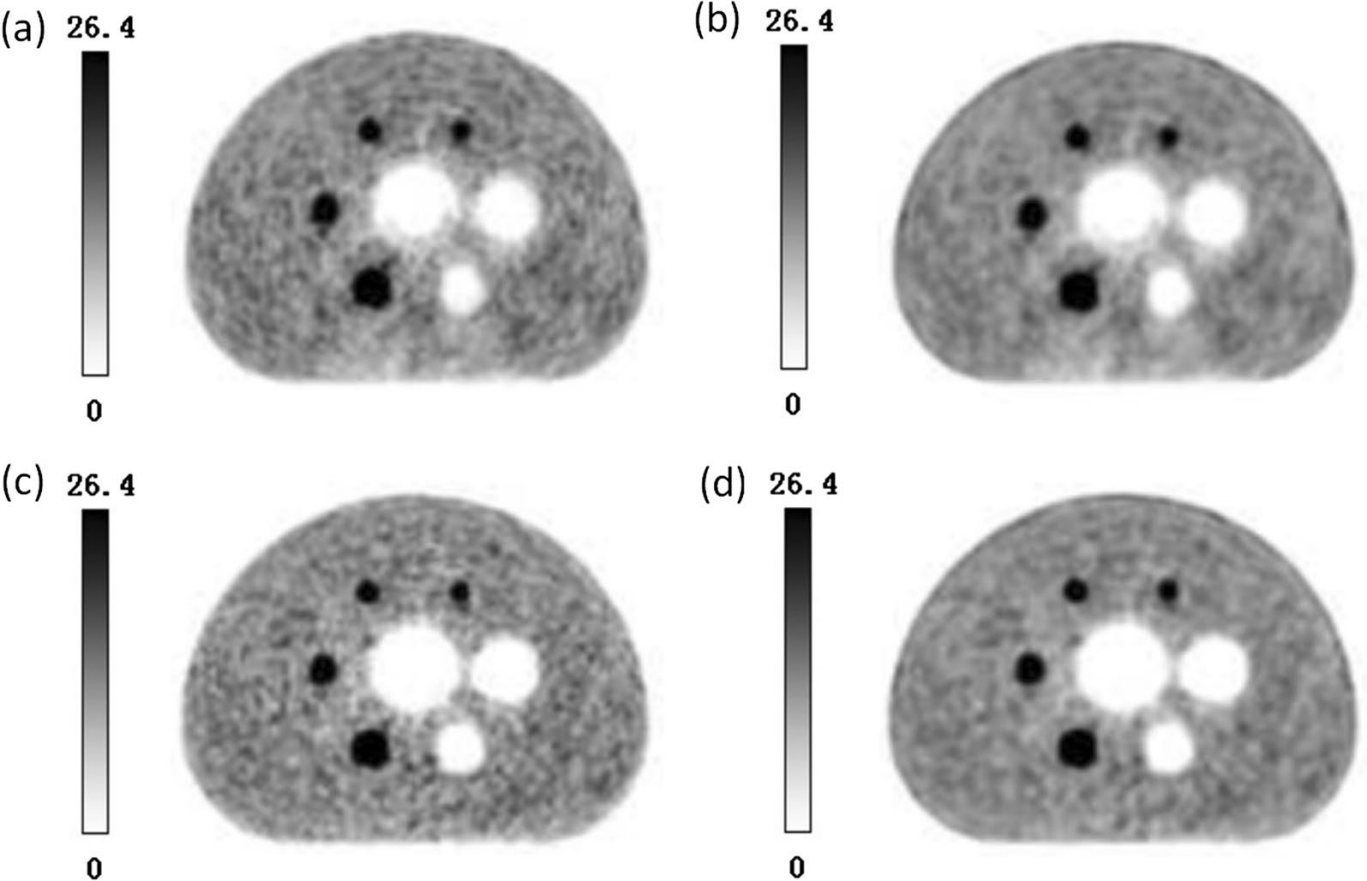
PET images for (**a**) OSEM, (**b**) OSEM-PSF, (**c**) OSEM-TOF and (**d**) OSEM-PSF-TOF

In order to compare the image quality of NIM PET/CT phantom under different reconstruction algorithms, four different algorithms (OSEM, OSEM-PSF, OSEM-TOF, and OSEM-PSF-TOF) were applied to reconstruct PET images with a PET/CT system (Fig. 13). It was revealed that PSF-TOF significantly improved the resolution and the contrast of spheres. However, when only PSF was used, the edge of spheres remained vague. Besides, TOF effectively improved the edge of spheres, reduced noise level, and detected more details for imaging.

**Fig. 14:**
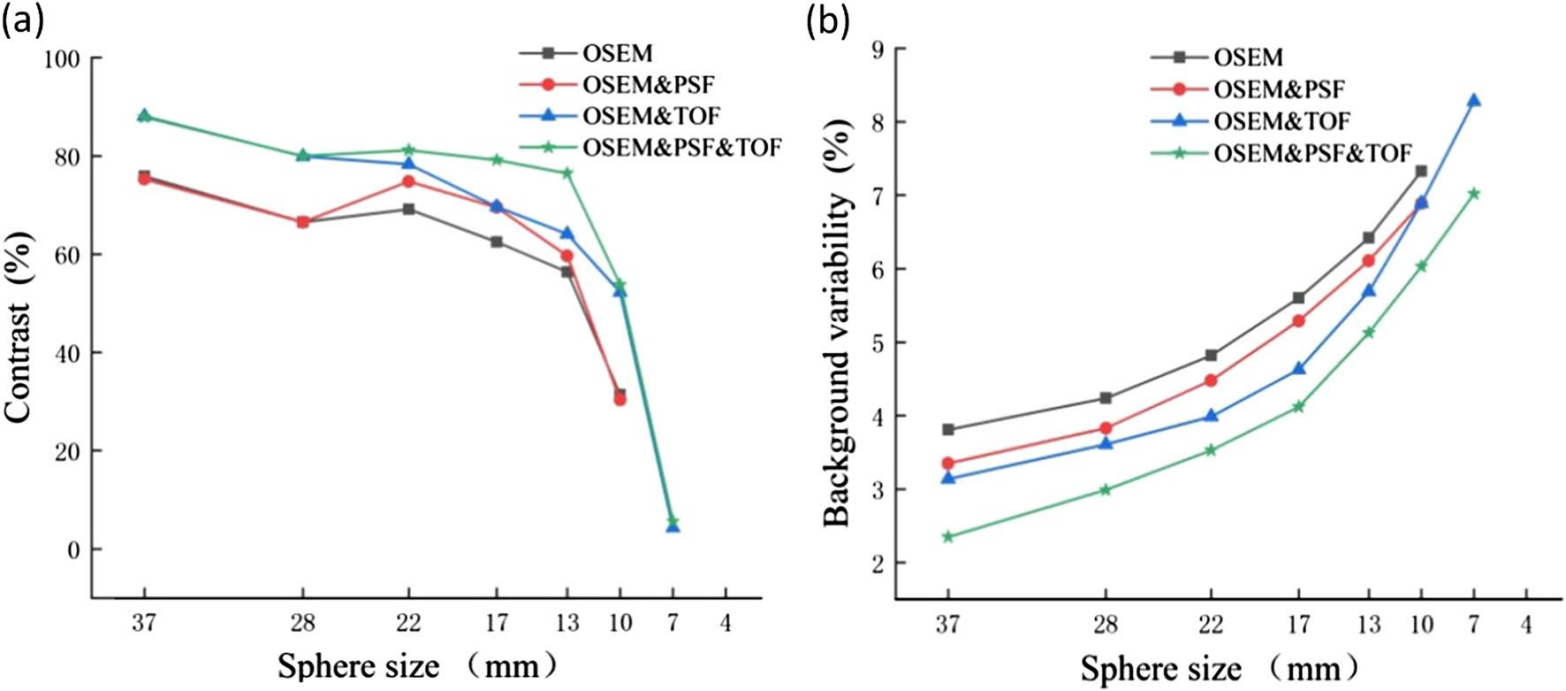
Image quality for spheres with different sizes versus (**a**) contrast and (**b**) background variability

In order to assess the level of improvement of image quality achieved by a reconstruction algorithm, the image contrast and background variability were calculated, presented in Table 8, and plotted as a linear graph (Fig. 14). It was found that PSF reduced the noise level and enhanced the image contrast. However, since PSF reconstruction exhibited to reduce the speed of convergence [22], the contrast and background variability of spheres (13–22 mm only) were significantly improved after two iterations. In terms of 37 mm, 28 mm and 10 mm spheres, PSF influences little on image contrast. In addition to improve the image contrast and background variability, TOF showed to greatly elevate the overall image quality and instrument detection limit. TOF-PSF could noticeably improve quality of imaging, as the diameter of the sphere decreases, this improvement becomes greater, and the largest increase for image contrast occurred in 10 mm sphere, reaching 22.4% (Bold numbers in Table 8).

**Table 8:** Image quality parameters for four different algorithms

Sphere size (mm)	Contrast (%)				Background variability (%)			
	OESM-PSF-TOF	OSEM-TOF	OSEM-PSF	OSEM	OESM-PSF-TOF	OSEM-TOF	OSEM-PSF	OSEM
37	87.87	88.06	75.25	75.86	2.35	3.14	3.35	3.81
28	79.99	79.88	66.51	66.52	2.99	3.61	3.83	4.24
22	81.16	78.31	74.8	69.14	3.53	3.99	4.48	4.82
17	79.17	69.63	69.53	62.48	4.12	4.63	5.29	5.6
13	76.44	64.12	59.66	56.38	5.13	5.69	6.11	6.42
10	**53.81**	52.27	30.3	**31.41**	6.03	6.89	6.89	7.33
7	5.61	4.32	–	–	7.02	8.28	–	–
4	–	–	–	–	–	–	–	–

Numerical results in bold represent the largest increase for image contrast occurred in 10mm sphere

As shown in Table 8, applying PSF and TOF simultaneously could effectively improve the contrast of an image and reduce the background variability.

## Discussion

The principle of PET imaging is the detection of gamma rays, originating from the annihilation of positrons with electrons within the examined object. Positron emitters with short half‐life are labelled to specific biological molecules and injected into patients. Depending on the carrier molecule, the radioisotope is distributed across different body tissues, providing physiological information from the ROI. Therefore, a critical requirement for designing a PET imaging phantoms is the feasibility to simulate radiotracer activity similar to that expected in clinical PET studies.

The present study proposed the PET/CT phantom for evaluating the PET image quality of micro-lesions, which is composed of a PET imaging module and a CT imaging module, and it can simultaneously detect the quality of PET/CT images. The minimum detectability is one of the most important tasks in a PET system, which is directly associated with the early diagnosis and staging of lesions [23, 24]. The minimum inner diameter of spheres used to measure parameters related to image quality in the NEMA IEC Body phantom is 10 mm according to the international guidelines [10]. The physical size of a lesion is practically difficult to derive from PET images due to spill-out and partial volume effects. Thus, in clinical studies, the quantification is generally based on the maximum voxel value or the mean value inside a three-dimensional (3D) contour. Oen et al. presented a PET image quality phantom with hot spheres, ranging from 4 to 20 mm in diameter, with sphere-to-background activity concentrations of 8:1 and 4:1, to mimic clinical conditions, and they found a similar detectability for the PET/MRI and the PET/CT [25]. Adler et al. developed a phantom in combination with an imaging protocol to detect micro lesions on different PET systems. Seven small spheres with inner diameters ranging from 3.95 to 15.43 mm were imaged [10]. Generally, diameters of spheres are totally different, making a serious challenge to compare the quality of imaging by the NEMA IEC Body phantom. Thus, phantoms are not fully compatible with the NEMA standard, and their validity cannot be fully confirmed. Raylman et al. assessed the capabilities and limitations of FDG-PET for detecting small tumors and lymph nodes, and found that PET with an attenuation correction consistently detected tumors with the size of not less than 9 mm [26]. Kadrmas et al. evaluated the effects of TOF on detection and localization of focal lesions in noisy PET images. According to their findings, TOF-PET provided a significant improvement in detecting focal lesions in a noisy background. The improvement in image quality can be utilized to clinically detect lesions and stage diseases [27]. Hashimoto et al. investigated the detectability of sub-centimeter spheres using a clinical PET/CT scanner. They used a clinical PET/CT scanner to obtain the data of a NEMA body phantom, consisting of 6 small spheres (inner diameters of 4.0, 5.0, 6.2, 7.9, 10, and 37 mm), containing ^18^F-FDG solution. The background activity was 2.65 kBq/mL, and the sphere-to-background ratio was 8. They found that the TOF with 2 mm voxels improved the detectability of sub-centimeter hot spheres on a clinical PET/CT scanner [28]. Although there are two spheres with the same size as the spheres of the NEMA IEC Body phantom, the results are not reliable due to the high level of noise. Therefore, the mentioned phantom is still only used for evaluating the image quality and relative detection limit of PET. Hence, all the phantoms used in the above-mentioned studies cannot be employed for assessment of PET systems. In the present study, the NIM PET/CT phantom increased the size of spheres (4 and 7 mm), and the combination of the PET phantom and CT phantom could remarkably reduce the time required for quality control of PET/CT images.

In order to verify the effectiveness of the NIM PET/CT phantom, in the current study, PET imaging was carried out by the NEMA IEC Body phantom and the NIM PET/CT phantom under the same scanning conditions. As illustrated in Fig. 7, spheres (10–28 mm) could be clearly observed with a great contrast, and a sphere (7 mm) could be detected by the NIM PET/CT phantom. It was revealed that the image quality and the residual error in the lung insert of the NIM PET/CT phantom were similar to those in the NEMA IEC Body phantom (Table 3). Thus, the NIM PET/CT phantom could not only realize the measurement of image quality and accurately estimate the residual error using the CT-based attenuation and scatter-corrected PET images, but also its results were comparable with those of the NEMA IEC Body phantom.

Additionally, the NIM PET/CT phantom was utilized with the three different PET/CT systems to perform scanning on the same scanning protocol, and Fig. 8 and Table 4 show that spheres (7 and 4 mm) could be detected, but 4 mm sphere is not visible in Fig. 7 due to the low contrast (3.22%). Besides, image quality, ability to detect micro lesions and accuracy of estimating the residual error using the CT-based attenuation and scatter-corrected PET images of “C” system were higher than those of “A” and “B” systems. Additionally, “B” system presents slightly better on cold lesions than “A” system. In terms of macro hot lesions, system “A” performs better than system “B”, but “B” system is far better to detect micro lesions than “A” system. Thus, the proposed phantom could reliably evaluate the quality of PET/CT images for detecting micro lesions.

CT imaging module contains low-contrast module and purified water, the results illustrate that the CT imaging module can evaluate low-contrast resolution of 1.5%, 1.0% and 0.5%. Furthermore, the background of low-contrast module is almost integrated with purified water due to the good water-equivalent characteristic. Therefore, a large area of the uniform CT image is gained to calculating CT image uniformity, and the CT values at the boundary were found similar and relatively stable. The uniformity of CT under three modes were calculated, and the results are similar.

Image contrast can be affected by uncertainties such as sampling techniques and noising [29]. PET images are subject to different noises while taking the data, which affects quality and diagnostics of the image. Noises reduce the quality of the image that badly disturbs the work of analyzing and processing image [30]. Therefore, eliminating the noise from the PET image is significant. So far, scientific research has adopted fuzzy preprocessing techniques to reduce the noise and enhance contrast, which has produced many good results [29]. Gaussian blur is a type of fuzzy preprocessing technique, which uses Gaussian filter as the smoothing filter to reduce the noise in PET images, but it is not enough for clinical diagnostics [16]. Thus, PSF and TOF techniques will be used to improve image resolution and enhance contrast. Table 8 illustrates that PSF could significantly improve the contrast for hot lesions, especially for spheres with diameters of 22 and 17 mm, whereas it could not noticeably improve the contrast for cold lesions, small hot lesions(10 mm) and was not efficacious for detecting micro-lesions (7 mm and 4 mm). Figures 13 and 14 show that PSF can reduce the noise of background, which cause the background image resolution improvement. Furthermore, when PSF was used only, the edge clarity of lesions were slightly ameliorated but remained vague. Therefore, PSF could slightly improve the detection of micro-lesions and image quality. In addition, PSF exhibited to effectively reduce the background variability and improve the image resolution [22]. TOF has shown a faster convergence with a comparable signal-to-noise ratio, as well as enhancing sharpening of edges in radiographic images, and improving the image contrast and background variability [16, 17], thereby enhancing the image resolution and detectability of PET/CT for micro-lesions [20, 27, 31, 32]. Figures 13 illustrates that TOF is not significant for the improvement of the background image resolution, but effectively improved the edge of lesions and enhance the contrast. TOF-PSF combines the advantages of both PSF and TOF, and it can significantly reduce the noise level and simultaneously enhance imaging details [22, 27], effectively decrease the background variability, enhance lesions contrast especially hot lesions, and improved lesions edge clarity. Thus, in follow-up research, combination of TOF with another reliable algorithm can be highly advantageous [20, 33], with supplementation of CT-based attenuation and scatter-corrected PET images [12], so as to effectively select an appropriate PET/CT system, accompanying with more details for medical imaging.

## Conclusions

In summary, in comparison with the NEMA IEC Body phantom, the NIM PET/CT phantom outperformed in evaluating the PET image quality of micro-lesions and performance parameters of the CT. However, concerning shortcoming of the proposed phantom, further research needs to be carried out to eliminate those shortcomings and achieve more reliable outcomes.

## Data Availability

The datasets generated during and/or analyzed during the current study are available from the corresponding author on reasonable request.

## Abbreviations

NEMA: National Electrical Manufacturers Association
PET: positron emission tomography
IEC: International Electrotechnical Commission
CT: computed tomography
NIM: National Institute of Metrology
^18^F-FDG: ^18^F-fluorodeoxyglucose
TOF: time-of-flight
OSEM: ordered subset expectation maximization
PSF: point spread function
2D: two-dimensional
ROI: region of interest
CRC: contrast recovery coefficient
SD: standard deviation
3D: three-dimensional
